# Improving reliability and validity in hip-hop dance assessment: Judging standards that elevate the sport and competition

**DOI:** 10.3389/fpsyg.2022.934158

**Published:** 2022-10-10

**Authors:** Nahoko Sato

**Affiliations:** Department of Physical Therapy, Faculty of Rehabilitation Science, Nagoya Gakuin University, Nagoya, Aichi, Japan

**Keywords:** hip-hop dance, judging system, aesthetic sport, reliability, validity, competition, Japan

## Abstract

This study examined the reliability and validity of judging system scores of past hip-hop dance competitions in Japan. The analysis focused on the scores for each assessment category separately. Judges’ scores were obtained from national dance competitions held annually in Japan between 2014 and 2019. In these competitions, five experienced judges evaluated the dancers’ performances. The judges scored on a 10-point scale in five categories as follows: creativity, expression and interpretation, impression, technical quality, and synchronisation. This study found that the technical quality category demonstrated good reliability, whilst the impression showed poor reliability. Systematic bias was significant for all categories. There are no levels of difficulty defined for technique, no criteria set for correct movement and no explanation provided for each scoring level, which suggests that each judge may have interpreted the criteria for evaluating hip-hop dance differently. Developing these definitions and identifying the biases that affect evaluation would ensure a reliable evaluation system.

## Introduction

Hip-hop dance is freestyle dance that began as street dancing, a part of the hip-hop culture (Craine and Mackrell, 2010), which includes breaking, rocking, popping, house and street jazz dances (Ojofeitimi et al., 2012). It has spread rapidly and many hip-hop dance competitions have been held worldwide. Originally, the impression of the audience was considered to be the most important factor in evaluating hip-hop dance; the winner of a competition was determined based on the audience’s extent of excitement. However, in recent years, hip-hop dance has become more competitive. It was first considered an Olympic sport in the 2018 Youth Olympics and will make its debut in the 2024 Olympics (International Olympic Committee, 2021). In this context, clear evaluation criteria must be defined for hip-hop dance to be considered a viable competition so that dancers, judges and audiences share a common understanding of the definition of superior hip-hop dance performance.

In Olympic artistic gymnastics, evaluations are divided into artistic and technical categories. Scores are determined by absolute evaluations that are based on the difficulty and kinematic criteria for all techniques, as defined in the Code of Points (Fédération Internationale de Gymnastique, 2021). Many studies have examined the reliability of this evaluation system using the results of past competitions, and high reliability has been reported (Leskošek et al., 2010; Atiković et al., 2011; Bučar et al., 2012; Pajek et al., 2013, 2014). In figure skating, another Olympic sport, final scores are calculated based on scores for technical elements, programme components and any deductions (International Skating Union, 2021). The reliability of figure-skating judges has also been investigated. Inter-judge correlation has been found to be above 0.9 for both technical and artistic scores (Lockwood et al., 2005). Thus, both artistic gymnastics and figure-skating competitions employ highly reliable evaluation systems.

In Dancesport, competitive ballroom dancing, a new evaluation system based on absolute evaluation, was introduced in 2013; this replaced the previous evaluation system that was found to be relative (World DanceSport Federation, 2021b). In the new evaluation system, as in artistic gymnastics and figure skating, evaluation is divided into artistic and technical aspects. The scoring system is based on a 10-point scale, with a performance description defined for each level. Research on the reliability of the new evaluation system reported that the mean correlation amongst all judges was 0.48 (Premelč et al., 2019), which was lower than correlation scores for artistic gymnastics and figure-skating competitions. Insufficient description of performance at each level was determined to be a reason for poor reliability.

At the biggest hip-hop dance competitions worldwide, multi-member groups compete, and their performances are evaluated across 10 categories in two domains (Table 1; Hip Hop International, 2021). The combined scores of the 10 categories are used to rank the competitors. Although descriptions of each category’s evaluations have been publicised, detailed kinematic criteria for techniques and the criteria for assessing each level along a scale have not been described. Thus, judges are likely to score dancers based on their own interpretations and criteria. At the 2018 Youth Olympic Games, all break-dancing (a form of hip-hop dance) matches were set up in a battle format, either individual or group, and the winner was determined by a relative evaluation based on which dancer was better in each of the six categories in three domains (Table 2; World DanceSport Federation, 2021a).

**Table 1:** Ten categories used in the most well-known hip-hop dance competitions.

Domain	Category
Performance	Creativity
	Staging, spacing, formations, and level changes
	Showmanship: intensity, confidence, projection and presence
	Style presence and attire
	Entertainment value/audience appeal
Skill	Musicality
	Synchronisation/timing
	Execution/controlled mobility and stabilisation
	Difficulty of execution of authentic dance style
	Variety of dance styles

**Table 2:** Six categories used to evaluate hop-hop dance performance at the 2018 Youth Olympic Games.

Domain	Category
Physical Quality: represents the qualities related to the body	Technique
	Variety
Interpretative Quality: represents the qualities related to the Soul	Performativity
	Musicality
Artistic Quality: represents the qualities related to the Mind	Creativity
	Personality

As in figure skating and artistic gymnastics, in hip-hop dance competitions, including break-dancing, performances have been evaluated in categories that include both technical and artistic aspects (Tables 1, 2). For the technical aspect of the assessment, difficulty levels for techniques have not been established, and the correct movements for each technique have not been defined; thus, it is not clear how judges evaluate the technical aspect of performance. Studies have reported that factors such as facial expression (Cunningham et al., 1990) and body shape (Tovée et al., 1999; Pawlowski et al., 2000), as well as movement, affect the judges’ evaluation of dance performances. Sato and Hopper (2021) found that the reliability of the judges’ scores varied when the actual dancer videos and humanoid animations created from actual dancer movements were evaluated, suggesting that dancer appearance impacted the evaluation of judges. Although several categories exist within the evaluation of the artistic aspect of hip-hop dancing in the current system (Table 1), evaluation categories that consider biases such as those (un) favouring facial expression or body shape have not been developed. To date, the reliability of the evaluation systems currently in use has not been reported based on the results of past competitions in hip-hop dance. To develop an objective evaluation system, the reliability of current evaluation systems must first be examined.

This study analysed judges’ scoring of hip-hop dance competitions held in the past, ascertaining each judging category separately and examining the reliability of the scores.

## Materials and methods

Judges’ scores were obtained from national dance competitions held annually in Japan throughout the years 2014–2019. However, the performances in these competitions were not videotaped. These competitions were open to dancers of elementary to junior high school age, and the results for each year, of the competition final, performed by the dance teams that won the preliminary rounds, were used for analysis. The dance team consisted of at least 5–40 dancers. Dance genres covered in this competition were hip-hop, which includes rocking, popping, breaking, house and street jazz. This study was approved by the Nagoya Gakuin University Research Ethics Committee. All data used in the analysis were anonymised, and participants were offered opt-out opportunities.

Five experienced judges evaluated the dancers’ performances in each competition. They were not the same individuals each year. The judges scored on a 10-point scale in five categories, as follows: creativity, expression and interpretation, impression, technical quality and synchronisation. There were no descriptions of performance for each point level (0–10), and the judges were not allowed to share or discuss their evaluations with each other. The final scores for each of the five categories for individual dance teams were calculated as the mean of the five judges’ scores.

Descriptive statistics of all judges’ scores for each category were calculated for each year of the competition. The following statistics values were calculated for validity analysis (Bučar et al., 2012). Signed and absolute deviations from the final score for individual judges were calculated as measures of bias. Mean rank and deviation from the expected rank were also assessed for individual judges. The expected rank was calculated as (*m* + 1)/2, where *m* is the number of judges, with reference to Bučar et al. (2012). The reliability of the evaluation was examined and assessed using intra-class correlation coefficients (ICC) for single and mean of five raters for both two-way random (consistency) and fixed (agreement) effects (Premelč et al., 2019). Kendall’s W (Kendall’s coefficient of concordance) was also calculated. A Kendall’s value of *W* < 0.40 was considered poor, 0.40–0.50 moderate, 0.50–0.70 good and greater than 0.70 excellent. ICC values were interpreted as follows: less than 0.40 poor reliability; 0.4–0.75 good reliability; greater than 0.75 excellent reliability (Fleiss et al., 2013). All data were analysed using SPSS Statistics software (version 25.0; SPSS Inc., Chicago, IL, United States).

## Results

Amongst the five categories, the highest mean score was 7.35 ± 1.03, for impression, and the lowest was 7.10 ± 1.13, for technical quality (Table 3). Appendix 1 presents the statistics of scores for individual judges, and Table 4 shows values extracted from them, indicating the best and worst deviations in judging. In terms of score bias, the maximum absolute deviation from the final score and mean rank deviation from the expected rank were generally significant for all categories. Regarding the correlation between the scores of the individual judges and the final score, which is the mean of the five judges, technical quality demonstrated the largest maximum correlation coefficient and impression demonstrated the smallest minimum correlation coefficient in most of the competition years.

**Table 3:** Mean, minimum and maximum values for five categories and the final scores for the 2015–2019 competitions.

			Mean		*SD*			The lowest marks			The highest marks			
Year	*n*	Category	Mean	Max	Min	Mean	Max	Min	Mean	Max	Min	Mean	Max	Min
2019	48	Creativity	7.64	8.65	6.69	1.31	1.64	0.98	4.80	6.00	4.00	9.60	10.00	9.00
		Expression and interpretation	7.61	8.67	6.56	1.17	1.75	0.65	5.00	7.00	3.00	9.60	10.00	9.00
		Impression	7.74	8.92	7.02	1.23	1.57	1.00	4.80	6.00	4.00	9.60	10.00	9.00
		Technical quality	7.64	8.27	7.08	1.37	2.05	0.83	4.80	6.00	3.00	9.80	10.00	9.00
		Synchronisation	7.79	9.19	6.83	1.18	1.55	0.81	4.80	6.00	3.00	9.60	10.00	9.00
2018	49	Creativity	7.11	7.73	6.65	0.92	1.19	0.63	5.00	6.00	4.00	8.80	9.00	8.00
		Expression and interpretation	7.11	7.92	6.31	0.91	1.16	0.53	5.20	7.00	4.00	8.80	9.00	8.00
		Impression	7.43	7.94	6.90	0.91	1.26	0.54	5.60	7.00	5.00	9.20	10.00	9.00
		Technical quality	7.19	7.98	6.63	0.98	1.27	0.53	5.20	7.00	4.00	9.00	10.00	8.00
		Synchronisation	7.27	8.18	6.67	0.80	1.07	0.51	5.60	7.00	4.00	8.60	9.00	8.00
2017	53	Creativity	7.12	7.72	6.57	1.07	1.41	0.74	5.20	6.00	5.00	9.20	10.00	8.00
		Expression and interpretation	7.00	7.83	6.38	0.98	1.48	0.64	5.20	7.00	4.00	9.20	10.00	8.00
		Impression	7.35	8.02	6.83	1.03	1.48	0.71	5.80	7.00	5.00	9.20	10.00	8.00
		Technical quality	7.01	7.85	6.06	1.04	1.33	0.60	5.40	7.00	5.00	9.20	10.00	8.00
		Synchronisation	7.22	8.00	6.45	0.88	1.26	0.62	5.80	7.00	5.00	9.00	10.00	8.00
2016	52	Creativity	6.73	6.96	6.40	1.15	1.50	0.96	4.60	5.00	4.00	9.40	10.00	9.00
		Expression and interpretation	6.71	7.25	6.23	0.95	1.33	0.52	4.80	6.00	4.00	8.60	10.00	8.00
		Impression	6.92	7.17	6.83	1.01	1.48	0.83	5.00	6.00	4.00	9.00	10.00	8.00
		Technical quality	6.61	7.50	5.83	1.17	1.49	0.78	4.40	5.00	4.00	9.00	10.00	8.00
		Synchronisation	6.95	7.46	6.42	1.00	1.44	0.73	5.20	6.00	4.00	9.00	10.00	8.00
2015	45	Creativity	6.95	7.60	6.24	1.02	1.48	0.67	5.20	6.00	4.00	9.00	10.00	8.00
		Expression and interpretation	7.14	7.96	6.13	0.99	1.46	0.66	5.20	6.00	4.00	9.20	10.00	8.00
		Impression	7.30	8.18	6.20	0.97	1.63	0.62	5.40	6.00	4.00	9.40	10.00	9.00
		Technical quality	7.05	7.87	6.02	1.11	1.62	0.76	5.00	6.00	4.00	9.40	10.00	9.00
		Synchronisation	6.95	7.78	6.13	0.96	1.31	0.63	5.20	6.00	4.00	9.00	10.00	8.00
Mean 2015–2019	247	Creativity	7.11	7.73	6.51	1.09	1.44	0.80	4.96	5.80	4.20	9.20	9.80	8.40
		Expression and interpretation	7.12	7.92	6.32	1.00	1.44	0.60	5.08	6.60	3.80	9.08	9.80	8.20
		Impression	**7.35**	8.05	6.76	1.03	1.49	0.74	5.32	6.40	4.40	9.28	10.00	8.60
		Technical quality	**7.10**	7.89	6.32	1.13	1.55	0.70	4.96	6.20	4.00	9.28	10.00	8.40
		Synchronisation	7.24	8.12	6.50	0.96	1.32	0.66	5.32	6.40	4.00	9.04	9.80	8.20

**Table 4:** The performance of individual judges.

			Absolute deviation		Judge mean rank deviation from the expected mean rank		Corrected category-5 judges mean correlation of individual judges	
Year	n	Category	min	max	min	max	min	max
2019	48	Creativity	0.68	1.35	−1.42	0.77	0.44	0.84
		Expression and interpretation	0.59	1.42	−1.52	0.96	0.57	0.83
		Impression	0.66	1.36	−1.67	0.42	0.42	0.75
		Technical quality	0.58	1.10	−1.25	0.35	0.76	0.90
		Synchronisation	0.57	1.66	−1.75	0.79	0.54	0.79
2018	49	Creativity	0.45	0.74	−1.61	0.18	0.53	0.76
		Expression and interpretation	0.57	0.95	−1.61	0.80	0.50	0.82
		Impression	0.49	0.80	−1.43	0.27	0.42	0.71
		Technical quality	0.56	0.89	−1.69	0.27	0.66	0.84
		Synchronisation	0.51	0.93	−1.88	0.43	0.25	0.85
2017	53	Creativity	0.56	0.82	−1.45	0.34	0.55	0.86
		Expression and interpretation	0.60	0.91	−1.58	0.45	0.46	0.85
		Impression	0.55	0.87	−1.42	0.17	0.41	0.83
		Technical quality	0.50	0.97	−1.49	0.96	0.55	0.88
		Synchronisation	0.64	0.96	−1.51	0.72	0.46	0.84
2016	52	Creativity	0.50	0.87	−0.98	−0.08	0.58	0.81
		Expression and interpretation	0.50	0.83	−1.25	0.17	0.42	0.79
		Impression	0.49	0.85	−1.08	−0.40	0.41	0.82
		Technical quality	0.64	0.94	−1.60	0.50	0.77	0.88
		Synchronisation	0.45	0.76	−1.31	0.23	0.56	0.85
2015	45	Creativity	0.52	1.09	−1.42	0.42	0.29	0.77
		Expression and interpretation	0.55	1.23	−1.56	0.96	0.50	0.85
		Impression	0.50	1.36	−1.62	0.84	0.17	0.82
		Technical quality	0.50	1.27	−1.60	0.98	0.68	0.84
		Synchronisation	0.50	1.08	−1.58	0.80	0.39	0.77

In terms of score reliability, the Kendall’s *W* values ranged from 0.319 to 0.681 (Table 5). In each year of the competition, the category with the highest reliability was technical quality, with most values indicating good reliability, with scores ranging from 0.576 to 0.681. The category with the lowest reliability was impression, with most values indicating poor reliability, with scores ranging from 0.319 to 0.448. Similar ICC results were obtained; the single-measure ICC coefficients for absolute agreement and consistency for technical quality demonstrated fair to good reliability. The average-measure ICC coefficients for absolute agreement and consistency for almost all categories showed good to excellent reliability.

**Table 5:** Reliability for the five categories and the final score for the 2015–2019 competitions.

Year	Category	ICC				Kendall’s W coefficient	
		Absolute agreement		Consistency			
		Single-measure (95%CI)	Average-measure (95%CI)	Single-measure (95%CI)	Average-measure (95%CI)	*W*	*p*
2019	Creativity	0.255 (0.129–0.409)	0.631 (0.426–0.776)	0.321 (0.193–0.472)	0.702 (0.545–0.817)	0.448	0.000
	Expression and interpretation	0.280 (0.136–0.446)	0.661 (0.440–0.801)	0.382 (0.251–0.531)	0.756 (0.626–0.850)	0.540	0.000
	Impression	0.174 (0.069–0.314)	0.514 (0.271–0.696)	0.229 (0.111–0.379)	0.598 (0.385–0.753)	0.385	0.000
	Technical quality	0.507 (0.366–0.648)	0.837 (0.743–0.902)	0.552 (0.425–0.681)	0.861 (0.787–0.914)	0.659	0.000
	Synchronisation	0.234 (0.096–0.398)	0.604 (0.347–0.768)	0.351 (0.222–0.502)	0.730 (0.587–0.834)	0.503	0.000
2018	Creativity	0.297 (0.172–0.447)	0.678 (0.510–0.801)	0.297 (0.172–0.447)	0.678 (0.51–0.801)	0.432	0.000
	Expression and interpretation	0.223 (0.092–0.381)	0.589 (0.337–0.755)	0.329 (0.202–0.479)	0.710 (0.559–0.821)	0.452	0.000
	Impression	0.173 (0.070–0.309)	0.511 (0.274–0.691)	0.197 (0.085–0.343)	0.551 (0.317–0.723)	0.364	0.000
	Technical quality	0.343 (0.181–0.513)	0.723 (0.525–0.841)	0.454 (0.323–0.595)	0.806 (0.705–0.880)	0.576	0.000
	Synchronisation	0.158 (0.054–0.296)	0.484 (0.222–0.677)	0.238 (0.12–0.387)	0.610 (0.406–0.759)	0.372	0.000
2017	Creativity	0.341 (0.204–0.493)	0.721 (0.561–0.829)	0.410 (0.284–0.549)	0.776 (0.665–0.859)	0.543	0.000
	Expression and interpretation	0.224 (0.100–0.374)	0.591 (0.358–0.749)	0.312 (0.189–0.457)	0.694 (0.539–0.808)	0.449	0.000
	Impression	0.234 (0.114–0.380)	0.604 (0.391–0.754)	0.305 (0.184–0.449)	0.687 (0.531–0.803)	0.448	0.000
	Technical quality	0.340 (0.152–0.526)	0.720 (0.472–0.847)	0.509 (0.383–0.639)	0.838 (0.756–0.898)	0.592	0.000
	Synchronisation	0.195 (0.071–0.347)	0.548 (0.276–0.727)	0.315 (0.194–0.459)	0.697 (0.546–0.809)	0.449	0.000
2016	Creativity	0.385 (0.260–0.527)	0.758 (0.637–0.848)	0.395 (0.268–0.537)	0.765 (0.647–0.853)	0.500	0.000
	Expression and interpretation	0.306 (0.180–0.453)	0.688 (0.524–0.805)	0.353 (0.228–0.498)	0.732 (0.597–0.832)	0.479	0.000
	Impression	0.238 (0.124–0.382)	0.610 (0.415–0.755)	0.239 (0.124–0.383)	0.612 (0.415–0.756)	0.399	0.000
	Technical quality	0.431 (0.232–0.610)	0.791 (0.602–0.887)	0.578 (0.458–0.698)	0.873 (0.808–0.920)	0.681	0.000
	Synchronisation	0.350 (0.215–0.500)	0.729 (0.578–0.833)	0.409 (0.282–0.550)	0.776 (0.663–0.859)	0.522	0.000
2015	Creativity	0.129 (0.035–0.263)	0.426 (0.152–0.641)	0.156 (0.046–0.305)	0.481 (0.195–0.687)	0.319	0.000
	Expression and interpretation	0.198 (0.079–0.351)	0.553 (0.300–0.730)	0.280 (0.152–0.437)	0.660 (0.473–0.795)	0.416	0.000
	Impression	0.117 (0.028–0.244)	0.399 (0.126–0.618)	0.170 (0.058–0.321)	0.507 (0.235–0.702)	0.319	0.000
	Technical quality	0.339 (0.165–0.521)	0.719 (0.497–0.845)	0.472 (0.336–0.617)	0.817 (0.716–0.890)	0.589	0.000
	Synchronisation	0.164 (0.059–0.306)	0.495 (0.238–0.688)	0.224 (0.104–0.379)	0.591 (0.366–0.753)	0.401	0.000

## Discussion

To develop hip-hop dance competition and elevate its competitive status, an evaluation system with high reliability must be developed. This study was the first to examine the reliability of evaluation results of hip-hop dance competitions.

Regarding the reliability, the Kendall’s *W* values ranged from 0.319 to 0.681, which was comparable to the reliability assessments for Dancesport (Premelč et al., 2019), thus indicating that the reliability was not high. In contrast, high reliability has been reported for judging in artistic gymnastics competitions (Leskošek et al., 2010; Atiković et al., 2011; Bučar et al., 2012; Pajek et al., 2013). In artistic gymnastics, the level of difficulty and correct movements for all techniques are defined, and point deductions are described in detail in the Code of Points. However, in hip-hop dance, there are no defined criteria for the difficulty of a technique or a correct movement, and there are no descriptions of each of the 10-point level. This means that each judge may interpret the criteria for evaluation and evaluate the performance differently in hip-hop dance. Various biases also reportedly affect judges’ evaluations, including the position of the judges (Dallas et al., 2011), experience of the judges (Flessas et al., 2015), order of the performances (Plessner, 1999) and reputation of the dancers (Findlay and Ste-Marie, 2004). Factors such as the dancers’ facial expression and appearance also affect performance evaluations (Cunningham et al., 1990; Tovée et al., 1999; Pawlowski et al., 2000; Sato and Hopper, 2021). These biases may have impacted the low reliability found in this study.

In hip-hop dance, dancers typically perform in groups. Similarly, rhythmic gymnastics involves a group competition, in which five competitors perform, and the judges must evaluate the performances of the five gymnasts simultaneously. The reliability of performance evaluations in artistic gymnastics and figure skating reported in previous studies were all for individual performance competitions, and no studies to date have investigated the reliability of performance evaluations in team competitions such as rhythmic gymnastics. When judges pay attention to one competitor, they lose information about execution to other competitors. Flessas et al. (2015) reported that when evaluating the five-gymnast ensemble routines in rhythmic gymnastics, international-level judges did not rely on eye fixation to detect errors and may have used other cognitive strategies, as compared to novice and national-level judges. Thus, evaluating performance in the case of group competitions can be considered more challenging, and this may also have affected the reliability results of hip-hop dance.

This study assessed systematic bias in judging to evaluate score validity. For all categories, the values of absolute deviations from the final score and mean rank and deviation from the expected rank were larger than those values for artistic gymnastics (Bučar et al., 2012), suggesting a more significant systematic bias. Fernandez-Villarino et al. (2013) reported that the special circumstances in which judges must evaluate dancers of different ages and skill levels in one competition could create problems, thereby making it difficult for judges to distinguish performances. The competitions analysed in this study were open to students from elementary to junior high school age; thus, a wide range of skill levels was likely observed and incorporated into performance evaluations. This wide range may be one of the reasons for the higher systematic bias that was found. Pajek et al. (2014) suggested that a possible reason for the low validity of artistic scores in artistic gymnastics was poorly defined criteria in the Code of Points. In this study, biases due to judges’ perceptions and preferences in evaluating the quality of performance and differences in interpretation of the judging criteria are assumed to contribute to score variability.

Amongst the evaluation categories used in this study, technical quality and synchronisation fall within the technical category, whilst creativity, expression/interpretation and impression fall within the artistic category. Technical quality, on the technical side, demonstrated the highest reliability, whilst impression, on the artistic side, showed the lowest reliability. Similar results were found in figure skating (Lockwood et al., 2005), artistic gymnastics (Pajek et al., 2014) and Dancesport (Premelč et al., 2019). These results implicate that the artistic side of evaluation may be more impacted by factors, including facial expression and body shape, as previous studies have demonstrated (Cunningham et al., 1990; Tovée et al., 1999; Pawlowski et al., 2000). Therefore, a new evaluation system that accounts for this effect would improve reliability on the artistic side of evaluation of hip-hop dance.

To implement a reliable evaluation system in hip-hop dance competitions, a detailed description of each level for each category must be provided as a first step. A clear evaluation system or tool will help judges interpret the criteria in the same way, thus reducing score variability due to differences in interpretation. Second, evaluation categories must also be reconsidered. In hip-hop dance, many factors other than movement are considered to affect performance evaluation. Evaluation categories should be based on the factors that affect performance evaluation. In artistic gymnastics and figure-skating competitions, rankings are determined by the final technical and artistic point scores. In hip-hop dance, the difficulty of the technique is important, but the artistic aspect is also important. The weight of the technical and artistic aspects in the evaluation, including the number of evaluation categories for each of these two aspects, must be considered. Third, biases that have been reported, including the order of performance, the position of the judges and the experience of the judges, should also be verified in hip-hop dance. Fourth, using a video system that is designed to record performances and observe them immediately afterwards would allow judges to observe dances multiple times; the use of such a system should be considered. Fifth, in hip hop dance competitions that are performed as a group competition, the evaluation criteria must be provided separately for individual dancers’ performance and group performance. In rhythmic gymnastics, the evaluation criteria are separately defined for the evaluation of individual gymnasts and collaborative performances (Fédération Internationale de Gymnastique, 2021).

In this study, the results of hip-hop dance competitions in which multiple dance groups’ performances are ranked by performance scores (similar to gymnastics and figure skating) were analysed. However, break dancers will most likely compete in a one-on-one battle format at the Paris Olympics. Break dancing originated in hip-hop culture, and the winner is determined by the extent of the audience’s excitement, which is influenced by their preferences and subjective impressions of the performance. However, as hip-hop dance (including breakdancing) has grown in popularity, objective evaluation systems have been developed to combat potential biases such as reputation and style preferences (Fogarty, 2018). Although the competition format for break dancing at the Paris Olympics is unknown, our findings can be used to develop a reliable standard of evaluation in a battle format. As mentioned earlier, multiple evaluation categories (divided into technical and artistic aspects) should be established, as well as a detailed description of each level for each category. Given that the characteristics of break dance are strongly linked to the creative expression of one’s identity, emotions and artistic sensibilities, the weightage of technical and artistic aspects should also be considered in the final score (Fogarty, 2018). Competing to determine which dancer is better scored in these evaluation categories allows for a more reliable evaluation.

This study has a few limitations. First, the performances of dancers with a wide range of skill levels were used for evaluation, as the competitions from which the data were pulled and analysed were open to elementary and junior high school-aged participants. Study results may have been different using data from competitions with more skilled adult dance performances. Second, only the scores of judges from competitions in Japan were analysed. Further studies should be undertaken to investigate scores from competitions held in other countries and world competitions. Third, it is not clear how the judges who participated in the competitions analysed in this study varied in their ability to evaluate the performance accurately and consistently. Since judges’ experience is an important factor influencing evaluation reliability (Flessas et al., 2015), this factor may have influenced this study’s results.

This study was the first to investigate the reliability of the evaluation results of hip-hop dance competitions. The study’s results will contribute to the development of a more reliable evaluation system for hip-hop dance competitions. To implement a reliable evaluation system, the reliability of the evaluation must be constantly investigated and feedback must be provided at the same time the system is developed. An evaluation system that can be explained objectively provides not only reliable evaluations but also guidelines for dancers and coaches to use as they work towards achieving high scores in competitions. A new evaluation system will ensure that hip-hop dance continues to develop as an Olympic sport.

## Data availability statement

The datasets generated and/or analyzed during the current study are not publicly available due to contract with the organisation that provided the data but are available from the corresponding author on reasonable request.

## Ethics statement

Studies involving human participants were reviewed and approved by the Nagoya Gakuin University Research Ethics Committee. Written informed consent from the (patients/ participants or patients/participants legal guardian/next of kin) was not required to participate in this study in accordance with the national legislation and the institutional requirements.

## Author contributions

NS contributed to the conception and design of the study, organized the database, performed the statistical analysis, and wrote the manuscript.

## Funding

This work was supported by the Nagoya Gakuin University Grant (2021–2024).

## Conflict of interest

The author declares that the research was conducted in the absence of any commercial or financial relationships that could be construed as a potential conflict of interest.

## Publisher’s note

All claims expressed in this article are solely those of the authors and do not necessarily represent those of their affiliated organizations, or those of the publisher, the editors and the reviewers. Any product that may be evaluated in this article, or claim that may be made by its manufacturer, is not guaranteed or endorsed by the publisher.

## References

[ref1] Atiković A. Kalinski S. D. Bijelić S. Vukadinović N. A. (2011). Analysis results judging world championships in men’s artistic gymnastics in the London 2009 year. Sport. Log. 7, 95–102. doi: 10.5550/sgia.110702.en.095A

[ref2] Bučar M. Čuk I. Pajek J. Karacsony I. Leskošek B. (2012). Reliability and validity of judging in women’s artistic gymnastics at university games 2009. Eur. J. Sport Sci. 12, 207–215. doi: 10.1080/17461391.2010.551416

[ref3] Craine D. Mackrell J. (2010). The Oxford Dictionary of Dance. New York: Oxford University Press.

[ref4] Cunningham M. R. Barbee A. P. Pike C. L. (1990). What do women want? facialmetric assessment of multiple motives in the perception of male facial physical attractiveness. J. Pers. Soc. Psychol. 59, 61–72. doi: 10.1037/0022-3514.59.1.61, PMID: 2213490

[ref5] Dallas G. Mavidis A. Chairopoulou C. (2011). Influence of angle of view on judges’ evaluations of inverted cross in men’s rings. Percept. Mot. Skills 112, 109–121. doi: 10.2466/05.22.24.27.PMS.112.1.109-121, PMID: 21466084

[ref6] Fédération Internationale de Gymnastique (2021). Rules. Available at: https://www.gymnastics.sport/site/rules (Accessed November 9, 2021).

[ref7] Fernandez-Villarino M. A. Bobo-Arce M. Sierra-Palmeiro E. (2013). Practical skills of rhythmic gymnastics judges. J. Hum. Kinet. 39, 243–249. doi: 10.2478/hukin-2013-0087, PMID: 24511360 PMC3916910

[ref8] Findlay L. C. Ste-Marie D. M. (2004). A reputation bias in figure skating judging. J. Sport Exerc. Psychol. 26, 154–166. doi: 10.1123/jsep.26.1.154

[ref9] Fleiss J. L. Levin B. Paik M. C. (2013). Statistical Methods for Rates and Proportions New Jersey: John Wiley & Sons.

[ref10] Flessas K. Mylonas D. Panagiotaropoulou G. Tsopani D. Korda A. Siettos C. (2015). Judging the judges’ performance in rhythmic gymnastics. Med. Sci. Sports Exerc. 47, 640–648. doi: 10.1249/MSS.0000000000000425, PMID: 24977695

[ref11] Fogarty M. (2018). “Why are breaking battles judged? The rise of international competitions” in The Oxford Handbook of Dance and Competition. ed. Dodds S. (New York: Oxford University Press), 409–428.

[ref12] Hip Hop International (2021). Rules Regul. Available at: http://www.hiphopinternational.com/officialrules/ (Accessed November 9, 2021).

[ref13] International Olympic Committee (2021). Breaking. Available at: https://olympics.com/en/sports/breaking/ (Accessed November 9, 2021).

[ref14] International Skating Union (2021). ISU judging system. Available at: https://www.isu.org/figure-skating/rules/fsk-judging-system (Accessed November 9, 2021).

[ref15] Leskošek B. Čuk I. Karácsony I. Pajek J. Bučar M. (2010). Reliability and validity of judging in men’s artistic gymnastics at the 2009 university games. Sci. Gymnast J. 2, 25–34.

[ref16] Lockwood K. L. McCreary D. R. Liddell E. (2005). Evaluation of success in competitive figure skating: an analysis of interjudge reliability. Avante 11, 1–9.

[ref17] Ojofeitimi S. Bronner S. Woo H. (2012). Injury incidence in hip hop dance. Scand. J. Med. Sci. Sports 22, 347–355. doi: 10.1111/j.1600-0838.2010.01173.x 20807386

[ref18] Pajek M. B. Cuk I. Pajek J. Kovač M. Leskošek B. (2013). Is the quality of judging in women artistic gymnastics equivalent at major competitions of different levels? J. Hum. Kinet. 37, 173–181. doi: 10.2478/hukin-2013-0038, PMID: 24146718 PMC3796836

[ref19] Pajek M. B. Čuk I. Pajek J. Kovač M. Leskošek B. (2014). The judging of artistry components in female gymnastics: a cause for concern? Sci. Gymnast J. 6, 5–12.

[ref20] Pawlowski B. Dunbar R. I. Lipowicz A. (2000). Tall men have more reproductive success. Nature 403:156. doi: 10.1038/35003107, PMID: 10646589

[ref21] Plessner H. (1999). Expectation biases in gymnastics judging. J. Sport Exerc. Psychol. 21, 131–144. doi: 10.1123/jsep.21.2.131

[ref22] Premelč J. Vučković G. James N. Leskošek B. (2019). Reliability of judging in dance sport. Front. Psychol. 10:1001. doi: 10.3389/fpsyg.2019.01001, PMID: 31133935 PMC6524706

[ref23] Sato N. Hopper L. S. (2021). Judges’ evaluation reliability changes between identifiable and anonymous performance of hip-hop dance movements. PLoS One 16:e0245861. doi: 10.1371/journal.pone.0245861, PMID: 33493189 PMC7833165

[ref24] Tovée M. J. Maisey D. S. Emery J. L. Cornelissen P. L. (1999). Visual cues to female physical attractiveness. Proc. Biol. Sci. 266, 211–218. doi: 10.1098/rspb.1999.0624, PMID: 10097394 PMC1689653

[ref25] World DanceSport Federation (2021a). Buenos Aires 2018 youth Olympic games rules and regulations. Available at: https://www.worlddancesport.org/News/BreakingForGold/BAYOG_Rules_and_Regulations-2667 (Accessed November 9, 2021).

[ref26] World DanceSport Federation (2021b). Judging systems. Available at: https://www.worlddancesport.org/Rule/Competition/General/Judging_Systems (Accessed November 9, 2021).

