# Supplementary material for: Improving reliability and validity in hip-hop dance assessment: Judging standards that elevate the sport and competition
Source: Front Psychol. 2022 Oct 10;13:934158. doi: 10.3389/fpsyg.2022.934158 (PMC9589446; doi:10.3389/fpsyg.2022.934158)
Supplement: Supplementary file 1 [file Table_1.DOCX]

**Appendix 1. Individual judges score statistics.**

**Table A 1.1. The statistics of judges’ score for the 2019 competition.**

| Category | Judge | Judge's score | | Signed deviations from the final score | | Absolute deviations from the final score | | Correlation between individual judge's score and final score | Mean rank | Deviation from the expected rank |
| --- | --- | --- | --- | --- | --- | --- | --- | --- | --- | --- |
|  |  | Mean | SD | Mean | SD | Mean | SD |  |  |  |
| Creativity | 1 | 6.69 | 1.06 | -0.95 | 1.04 | 1.20 | 0.75 | 0.44 | 3.77 | 0.77 |
|  | 2 | 8.65 | 1.64 | 1.00 | 1.18 | 1.35 | 0.74 | 0.72 | 1.58 | -1.42 |
|  | 3 | 7.63 | 1.45 | -0.02 | 0.97 | 0.73 | 0.62 | 0.76 | 2.58 | -0.42 |
|  | 4 | 7.65 | 1.41 | 0.00 | 0.82 | 0.68 | 0.45 | 0.84 | 2.48 | -0.52 |
|  | 5 | 7.60 | 0.98 | -0.04 | 0.86 | 0.69 | 0.51 | 0.59 | 2.67 | -0.33 |
| Expression and interpretation | 1 | 6.56 | 0.80 | -1.05 | 0.75 | 1.12 | 0.63 | 0.60 | 3.96 | 0.96 |
|  | 2 | 8.67 | 1.75 | 1.06 | 1.14 | 1.42 | 0.63 | 0.82 | 1.48 | -1.52 |
|  | 3 | 7.38 | 1.38 | -0.23 | 0.91 | 0.79 | 0.50 | 0.76 | 2.85 | -0.15 |
|  | 4 | 7.73 | 1.27 | 0.12 | 0.73 | 0.63 | 0.37 | 0.83 | 2.40 | -0.60 |
|  | 5 | 7.71 | 0.65 | 0.10 | 0.74 | 0.59 | 0.44 | 0.57 | 2.48 | -0.52 |
| Impression | 1 | 7.02 | 1.12 | -0.72 | 1.06 | 1.06 | 0.71 | 0.42 | 3.42 | 0.42 |
|  | 2 | 8.92 | 1.27 | 1.18 | 1.03 | 1.36 | 0.77 | 0.58 | 1.33 | -1.67 |
|  | 3 | 7.71 | 1.57 | -0.03 | 1.11 | 0.92 | 0.61 | 0.75 | 2.60 | -0.40 |
|  | 4 | 7.38 | 1.20 | -0.36 | 0.82 | 0.73 | 0.52 | 0.73 | 3.08 | 0.08 |
|  | 5 | 7.67 | 1.00 | -0.07 | 0.82 | 0.66 | 0.48 | 0.59 | 2.63 | -0.38 |
| Technical quality | 1 | 7.08 | 1.16 | -0.56 | 0.72 | 0.74 | 0.52 | 0.81 | 3.35 | 0.35 |
|  | 2 | 8.27 | 2.05 | 0.63 | 1.13 | 1.10 | 0.66 | 0.90 | 1.75 | -1.25 |
|  | 3 | 7.38 | 1.38 | -0.27 | 0.89 | 0.78 | 0.51 | 0.76 | 2.79 | -0.21 |
|  | 4 | 7.81 | 1.44 | 0.17 | 0.72 | 0.58 | 0.46 | 0.87 | 2.10 | -0.90 |
|  | 5 | 7.67 | 0.83 | 0.03 | 0.74 | 0.62 | 0.39 | 0.77 | 2.48 | -0.52 |
| Synchronisation/timing | 1 | 6.83 | 0.97 | -0.95 | 0.88 | 1.10 | 0.69 | 0.54 | 3.79 | 0.79 |
|  | 2 | 9.19 | 1.55 | 1.40 | 1.08 | 1.66 | 0.59 | 0.75 | 1.25 | -1.75 |
|  | 3 | 7.48 | 1.24 | -0.31 | 0.83 | 0.71 | 0.53 | 0.74 | 2.85 | -0.15 |
|  | 4 | 7.60 | 1.32 | -0.18 | 0.83 | 0.67 | 0.52 | 0.79 | 2.79 | -0.21 |
|  | 5 | 7.83 | 0.81 | 0.05 | 0.68 | 0.57 | 0.36 | 0.66 | 2.31 | -0.69 |

**Table A 1.2. The statistics of judges’ score for the 2018 competition.**

| Category | Judge | Judge's score | | Signed deviations from the final score | | Absolute deviations from the final score | | Correlation between individual judge's score and final score | Mean rank | Deviation from the expected rank |
| --- | --- | --- | --- | --- | --- | --- | --- | --- | --- | --- |
|  |  | Mean | SD | Mean | SD | Mean | SD |  |  |  |
| Creativity | 1 | 6.65 | 0.80 | -0.45 | 0.70 | 0.69 | 0.46 | 0.55 | 3.18 | 0.18 |
|  | 2 | 6.82 | 0.97 | -0.29 | 0.68 | 0.56 | 0.48 | 0.72 | 2.80 | -0.20 |
|  | 3 | 7.20 | 1.19 | 0.10 | 0.82 | 0.64 | 0.52 | 0.76 | 2.24 | -0.76 |
|  | 4 | 7.73 | 1.02 | 0.63 | 0.71 | 0.74 | 0.59 | 0.72 | 1.39 | -1.61 |
|  | 5 | 7.12 | 0.63 | 0.02 | 0.61 | 0.45 | 0.41 | 0.53 | 2.31 | -0.69 |
| Expression and interpretation | 1 | 6.31 | 0.85 | -0.81 | 0.75 | 0.95 | 0.56 | 0.52 | 3.80 | 0.80 |
|  | 2 | 6.71 | 1.00 | -0.40 | 0.71 | 0.67 | 0.45 | 0.71 | 3.16 | 0.16 |
|  | 3 | 7.02 | 1.16 | -0.09 | 0.73 | 0.57 | 0.47 | 0.82 | 2.55 | -0.45 |
|  | 4 | 7.92 | 1.02 | 0.80 | 0.64 | 0.87 | 0.54 | 0.80 | 1.39 | -1.61 |
|  | 5 | 7.61 | 0.53 | 0.50 | 0.59 | 0.64 | 0.44 | 0.50 | 1.78 | -1.22 |
| Impression | 1 | 6.90 | 0.96 | -0.53 | 0.69 | 0.73 | 0.48 | 0.71 | 3.27 | 0.27 |
|  | 2 | 7.53 | 0.79 | 0.10 | 0.61 | 0.51 | 0.35 | 0.64 | 2.02 | -0.98 |
|  | 3 | 7.33 | 1.26 | -0.10 | 1.01 | 0.80 | 0.60 | 0.64 | 2.51 | -0.49 |
|  | 4 | 7.94 | 0.99 | 0.51 | 0.79 | 0.72 | 0.60 | 0.60 | 1.57 | -1.43 |
|  | 5 | 7.45 | 0.54 | 0.02 | 0.59 | 0.49 | 0.34 | 0.42 | 2.33 | -0.67 |
| Technical quality | 1 | 6.78 | 0.87 | -0.41 | 0.68 | 0.65 | 0.45 | 0.66 | 3.16 | 0.16 |
|  | 2 | 6.63 | 1.09 | -0.56 | 0.70 | 0.76 | 0.47 | 0.77 | 3.27 | 0.27 |
|  | 3 | 6.92 | 1.27 | -0.27 | 0.75 | 0.61 | 0.51 | 0.84 | 2.82 | -0.18 |
|  | 4 | 7.98 | 1.13 | 0.79 | 0.64 | 0.89 | 0.50 | 0.84 | 1.31 | -1.69 |
|  | 5 | 7.63 | 0.53 | 0.44 | 0.55 | 0.56 | 0.44 | 0.68 | 1.73 | -1.27 |
| Synchronisation/timing | 1 | 6.67 | 0.85 | -0.60 | 0.68 | 0.76 | 0.49 | 0.60 | 3.43 | 0.43 |
|  | 2 | 6.96 | 0.76 | -0.31 | 0.66 | 0.56 | 0.46 | 0.53 | 2.88 | -0.12 |
|  | 3 | 7.02 | 1.07 | -0.25 | 0.74 | 0.62 | 0.48 | 0.78 | 2.76 | -0.24 |
|  | 4 | 8.18 | 0.81 | 0.91 | 0.46 | 0.93 | 0.42 | 0.85 | 1.12 | -1.88 |
|  | 5 | 7.51 | 0.51 | 0.24 | 0.62 | 0.51 | 0.43 | 0.25 | 2.06 | -0.94 |

**Table A 1.3. The statistics of judges’ score for the 2017 competition.**

| Category | Judge | Judge's score | | Signed deviations from the final score | | Absolute deviations from the final score | | Correlation between individual judge's score and final score | Mean rank | Deviation from the expected rank |
| --- | --- | --- | --- | --- | --- | --- | --- | --- | --- | --- |
|  |  | Mean | SD | Mean | SD | Mean | SD |  |  |  |
| Creativity | 1 | 6.57 | 1.01 | -0.56 | 0.88 | 0.82 | 0.63 | 0.55 | 3.34 | 0.34 |
|  | 2 | 6.70 | 0.82 | -0.43 | 0.64 | 0.56 | 0.53 | 0.69 | 3.09 | 0.09 |
|  | 3 | 7.08 | 1.41 | -0.05 | 0.83 | 0.66 | 0.50 | 0.86 | 2.47 | -0.53 |
|  | 4 | 7.57 | 1.37 | 0.44 | 0.84 | 0.77 | 0.54 | 0.83 | 1.81 | -1.19 |
|  | 5 | 7.72 | 0.74 | 0.59 | 0.56 | 0.65 | 0.49 | 0.74 | 1.55 | -1.45 |
| Expression and interpretation | 1 | 6.46 | 0.80 | -0.66 | 1.10 | 0.83 | 0.97 | 0.46 | 3.27 | 0.27 |
|  | 2 | 6.38 | 0.77 | -0.62 | 0.56 | 0.65 | 0.52 | 0.71 | 3.45 | 0.45 |
|  | 3 | 6.81 | 1.23 | -0.19 | 0.82 | 0.60 | 0.58 | 0.77 | 2.70 | -0.30 |
|  | 4 | 7.53 | 1.48 | 0.53 | 0.96 | 0.91 | 0.61 | 0.85 | 1.85 | -1.15 |
|  | 5 | 7.83 | 0.64 | 0.83 | 0.62 | 0.86 | 0.57 | 0.58 | 1.42 | -1.58 |
| Impression | 1 | 6.83 | 0.89 | -0.52 | 0.77 | 0.75 | 0.53 | 0.57 | 3.17 | 0.17 |
|  | 2 | 7.00 | 0.83 | -0.35 | 0.58 | 0.55 | 0.39 | 0.73 | 2.87 | -0.13 |
|  | 3 | 6.91 | 1.48 | -0.45 | 0.98 | 0.86 | 0.64 | 0.83 | 2.94 | -0.06 |
|  | 4 | 8.02 | 1.25 | 0.67 | 0.85 | 0.87 | 0.63 | 0.76 | 1.70 | -1.30 |
|  | 5 | 8.00 | 0.71 | 0.65 | 0.77 | 0.83 | 0.57 | 0.41 | 1.58 | -1.42 |
| Technical quality | 1 | 6.83 | 1.09 | -0.18 | 0.61 | 0.50 | 0.39 | 0.83 | 2.68 | -0.32 |
|  | 2 | 6.06 | 0.89 | -0.95 | 0.54 | 0.97 | 0.50 | 0.81 | 3.96 | 0.96 |
|  | 3 | 6.62 | 1.27 | -0.39 | 0.77 | 0.70 | 0.49 | 0.82 | 3.02 | 0.02 |
|  | 4 | 7.71 | 1.33 | 0.56 | 1.22 | 0.95 | 0.94 | 0.88 | 1.52 | -1.48 |
|  | 5 | 7.85 | 0.60 | 0.84 | 0.71 | 0.95 | 0.56 | 0.55 | 1.51 | -1.49 |
| Synchronisation/timing | 1 | 6.77 | 0.75 | -0.45 | 0.72 | 0.69 | 0.47 | 0.46 | 2.96 | -0.04 |
|  | 2 | 6.45 | 0.64 | -0.77 | 0.51 | 0.77 | 0.50 | 0.67 | 3.72 | 0.72 |
|  | 3 | 6.87 | 1.14 | -0.35 | 0.71 | 0.64 | 0.46 | 0.84 | 2.96 | -0.04 |
|  | 4 | 8.00 | 1.26 | 0.78 | 0.82 | 0.96 | 0.59 | 0.83 | 1.55 | -1.45 |
|  | 5 | 8.00 | 0.62 | 0.78 | 0.61 | 0.83 | 0.55 | 0.51 | 1.49 | -1.51 |

**Table A 1.4. The statistics of judges’ score for the 2016 competition.**

| Category | Judge | Judge's score | | Signed deviations from the final score | | Absolute deviations from the final score | | Correlation between individual judge's score and final score | Mean rank | Deviation from the expected rank |
| --- | --- | --- | --- | --- | --- | --- | --- | --- | --- | --- |
|  |  | Mean | SD | Mean | SD | Mean | SD |  |  |  |
| Creativity | 1 | 6.40 | 1.00 | -0.33 | 0.73 | 0.63 | 0.49 | 0.69 | 2.92 | -0.08 |
|  | 2 | 6.60 | 1.50 | -0.13 | 1.01 | 0.87 | 0.52 | 0.76 | 2.77 | -0.23 |
|  | 3 | 6.96 | 1.24 | 0.23 | 0.81 | 0.69 | 0.46 | 0.76 | 2.02 | -0.98 |
|  | 4 | 6.87 | 1.05 | 0.13 | 0.62 | 0.50 | 0.38 | 0.81 | 2.04 | -0.96 |
|  | 5 | 6.83 | 0.96 | 0.10 | 0.83 | 0.63 | 0.55 | 0.58 | 2.23 | -0.77 |
| Expression and interpretation | 1 | 6.23 | 0.90 | -0.48 | 0.56 | 0.62 | 0.40 | 0.78 | 3.17 | 0.17 |
|  | 2 | 6.71 | 1.33 | 0.00 | 0.89 | 0.75 | 0.48 | 0.79 | 2.48 | -0.52 |
|  | 3 | 7.25 | 1.30 | 0.54 | 0.86 | 0.83 | 0.58 | 0.79 | 1.75 | -1.25 |
|  | 4 | 6.40 | 0.72 | -0.31 | 0.56 | 0.50 | 0.39 | 0.69 | 2.85 | -0.15 |
|  | 5 | 6.96 | 0.52 | 0.25 | 0.67 | 0.57 | 0.43 | 0.42 | 1.94 | -1.06 |
| Impression | 1 | 6.83 | 0.83 | -0.09 | 0.65 | 0.50 | 0.42 | 0.64 | 2.33 | -0.67 |
|  | 2 | 6.87 | 1.48 | -0.05 | 1.02 | 0.85 | 0.54 | 0.82 | 2.60 | -0.40 |
|  | 3 | 7.17 | 1.00 | 0.25 | 0.81 | 0.69 | 0.48 | 0.59 | 1.92 | -1.08 |
|  | 4 | 6.87 | 0.86 | -0.05 | 0.67 | 0.49 | 0.46 | 0.64 | 2.40 | -0.60 |
|  | 5 | 6.87 | 0.86 | -0.05 | 0.84 | 0.68 | 0.49 | 0.41 | 2.58 | -0.42 |
| Technical quality | 1 | 5.83 | 1.35 | -0.78 | 0.67 | 0.84 | 0.60 | 0.88 | 3.50 | 0.50 |
|  | 2 | 6.60 | 1.49 | -0.02 | 0.82 | 0.65 | 0.50 | 0.86 | 2.44 | -0.56 |
|  | 3 | 7.50 | 1.31 | 0.89 | 0.74 | 0.94 | 0.67 | 0.83 | 1.40 | -1.60 |
|  | 4 | 6.02 | 0.92 | -0.59 | 0.61 | 0.70 | 0.48 | 0.80 | 3.31 | 0.31 |
|  | 5 | 7.12 | 0.78 | 0.50 | 0.62 | 0.64 | 0.47 | 0.77 | 1.75 | -1.25 |
| Synchronisation/timing | 1 | 6.42 | 0.87 | -0.53 | 0.64 | 0.66 | 0.50 | 0.69 | 3.23 | 0.23 |
|  | 2 | 6.67 | 1.44 | -0.28 | 0.90 | 0.76 | 0.55 | 0.85 | 2.81 | -0.19 |
|  | 3 | 7.46 | 0.96 | 0.51 | 0.71 | 0.71 | 0.50 | 0.68 | 1.69 | -1.31 |
|  | 4 | 6.88 | 1.00 | -0.07 | 0.55 | 0.45 | 0.31 | 0.84 | 2.40 | -0.60 |
|  | 5 | 7.33 | 0.73 | 0.37 | 0.69 | 0.62 | 0.48 | 0.56 | 1.81 | -1.19 |

**Table A 1.5. The statistics of judges’ score for the 2015 competition.**

| Category | Judge | Judge's score | | Signed deviations from the final score | | Absolute deviations from the final score | | Correlation between individual judge's score and final score | Mean rank | Deviation from the expected rank |
| --- | --- | --- | --- | --- | --- | --- | --- | --- | --- | --- |
|  |  | Mean | SD | Mean | SD | Mean | SD |  |  |  |
| Creativity | 1 | 6.84 | 1.04 | -0.10 | 0.86 | 0.69 | 0.51 | 0.57 | 2.44 | -0.56 |
|  | 2 | 6.84 | 0.67 | -0.10 | 0.66 | 0.52 | 0.42 | 0.46 | 2.56 | -0.44 |
|  | 3 | 6.24 | 1.48 | -0.70 | 1.09 | 1.09 | 0.68 | 0.77 | 3.42 | 0.42 |
|  | 4 | 7.60 | 1.10 | 0.65 | 0.83 | 0.88 | 0.58 | 0.66 | 1.58 | -1.42 |
|  | 5 | 7.20 | 0.79 | 0.25 | 0.84 | 0.72 | 0.49 | 0.29 | 2.13 | -0.87 |
| Expression and interpretation | 1 | 7.13 | 1.01 | -0.01 | 0.85 | 0.68 | 0.51 | 0.56 | 2.49 | -0.51 |
|  | 2 | 6.98 | 0.69 | -0.16 | 0.67 | 0.55 | 0.41 | 0.52 | 2.78 | -0.22 |
|  | 3 | 6.13 | 1.46 | -1.01 | 0.96 | 1.23 | 0.64 | 0.85 | 3.96 | 0.96 |
|  | 4 | 7.96 | 1.15 | 0.81 | 0.75 | 0.96 | 0.56 | 0.78 | 1.44 | -1.56 |
|  | 5 | 7.51 | 0.66 | 0.37 | 0.67 | 0.62 | 0.45 | 0.50 | 1.96 | -1.04 |
| Impression | 1 | 7.24 | 1.03 | -0.06 | 0.82 | 0.68 | 0.44 | 0.61 | 2.69 | -0.31 |
|  | 2 | 7.42 | 0.62 | 0.12 | 0.62 | 0.50 | 0.38 | 0.48 | 2.42 | -0.58 |
|  | 3 | 6.20 | 1.63 | -1.10 | 1.19 | 1.36 | 0.87 | 0.82 | 3.84 | 0.84 |
|  | 4 | 8.18 | 0.94 | 0.88 | 0.69 | 0.96 | 0.55 | 0.68 | 1.38 | -1.62 |
|  | 5 | 7.47 | 0.63 | 0.16 | 0.79 | 0.67 | 0.44 | 0.17 | 2.29 | -0.71 |
| Technical quality | 1 | 7.20 | 1.08 | 0.15 | 0.64 | 0.50 | 0.43 | 0.80 | 2.24 | -0.76 |
|  | 2 | 6.64 | 0.98 | -0.40 | 0.69 | 0.64 | 0.47 | 0.73 | 3.18 | 0.18 |
|  | 3 | 6.02 | 1.62 | -1.03 | 1.03 | 1.27 | 0.71 | 0.82 | 3.98 | 0.98 |
|  | 4 | 7.87 | 1.12 | 0.82 | 0.62 | 0.87 | 0.54 | 0.84 | 1.40 | -1.60 |
|  | 5 | 7.51 | 0.76 | 0.46 | 0.66 | 0.68 | 0.42 | 0.68 | 1.82 | -1.18 |
| Synchronisation/timing | 1 | 6.87 | 1.16 | -0.08 | 0.91 | 0.70 | 0.58 | 0.62 | 2.67 | -0.33 |
|  | 2 | 6.69 | 0.63 | -0.26 | 0.57 | 0.50 | 0.37 | 0.59 | 2.80 | -0.20 |
|  | 3 | 6.13 | 1.31 | -0.81 | 0.98 | 1.08 | 0.66 | 0.71 | 3.80 | 0.80 |
|  | 4 | 7.78 | 1.04 | 0.83 | 0.69 | 0.94 | 0.53 | 0.77 | 1.42 | -1.58 |
|  | 5 | 7.27 | 0.65 | 0.32 | 0.70 | 0.62 | 0.45 | 0.39 | 2.02 | -0.98 |
